# Breaking down data silos across companies to train genome‐wide predictions: A feasibility study in wheat

**DOI:** 10.1111/pbi.70095

**Published:** 2025-04-20

**Authors:** Moritz Lell, Abhishek Gogna, Vincent Kloesgen, Ulrike Avenhaus, Jost Dörnte, Wera Maria Eckhoff, Tobias Eschholz, Mario Gils, Martin Kirchhoff, Michael Koch, Sonja Kollers, Nina Pfeiffer, Matthias Rapp, Valentin Wimmer, Markus Wolf, Jochen Reif, Yusheng Zhao

**Affiliations:** ^1^ Leibniz Institute for Plant Genetics and Crop Plant Research Seeland Germany; ^2^ W. von Borries‐Eckendorf GmbH & Co. KG Leopoldshöhe Germany; ^3^ Deutsche Saatveredelung AG Lippstadt Germany; ^4^ KWS SAAT SE & Co. KGaA Einbeck Germany; ^5^ Nordsaat Saatzucht GmbH Langenstein Germany; ^6^ KWS LOCHOW GmbH Northeim Germany; ^7^ SU BIOTEC GmbH Gatersleben Germany

**Keywords:** wheat, genomic prediction, data integration, big data, imputation

## Abstract

Big data, combined with artificial intelligence (AI) techniques, holds the potential to significantly enhance the accuracy of genome‐wide predictions. Motivated by the success reported for wheat hybrids, we extended the scope to inbred lines by integrating phenotypic and genotypic data from four commercial wheat breeding programs. Acting as an academic data trustee, we merged these data with historical experimental series from previous public–private partnerships. The integrated data spanned 12 years, 168 environments, and provided a genomic prediction training set of up to ~9500 genotypes for grain yield, plant height and heading date. Despite the heterogeneous phenotypic and genotypic data, we were able to obtain high‐quality data by implementing rigorous data curation, including SNP imputation. We utilized the data to compare genomic best linear unbiased predictions with convolutional neural network‐based genomic prediction. Our analysis revealed that we could flexibly combine experimental series for genomic prediction, with prediction ability steadily improving as the training set sizes increased, peaking at around 4000 genotypes. As training set sizes were further increased, the gains in prediction ability decreased, approaching a plateau well below the theoretical limit defined by the square root of the heritability. Potential avenues, such as designed training sets or novel non‐linear prediction approaches, could overcome this plateau and help to more fully exploit the high‐value big data generated by breaking down data silos across companies.

## Introduction

In the last decade, genome‐wide prediction has revolutionized plant breeding by providing an estimate of the genotypic value of a new candidate variety from its genomic profile and phenotype observations of related genotypes (Meuwissen *et al*., 2001). This allows some of the expensive field trials to be omitted, as early breeding trial stages with low observed heritability can be replaced using genomic prediction (Riedelsheimer and Melchinger, 2013). Thereby, the time required to select superior genotypes can be shortened (Beyene *et al*., 2021), even for complex traits controlled by many genes.

Given the large number of genes and their interactions that influence most agronomic traits, feasible population sizes in breeding programs do not allow us to infer the influence of each individual locus and locus interactions. Modern statistical techniques have been developed to address the shortcomings of traditional linear models in capturing complex gene interactions and genotype relationships, effectively reducing the influence of large amounts of noisy data (Chafai *et al*., 2023). The most practical and widely used methods are parametric or semi‐parametric models (Montesinos‐López *et al*., 2022). These models generally introduce *a priori* assumptions about the genetic effects, either by regularization of parameter estimates (de los Campos *et al*., 2013) or by selecting informative prior distributions in a Bayesian framework (Gianola, 2013). Originally, the parameters to be estimated were effects of genetic loci, as in Ridge‐Regression Best Linear Unbiased Prediction (rrBLUP, Meuwissen *et al*., 2001), but genetic effects of individuals can instead be modelled directly by including the expected correlation of their breeding values in the model. When pedigrees are unknown, these relationships can be inferred from genomic data, giving rise to Genomic BLUP (GBLUP, VanRaden, 2008). The resulting genomic kinship matrix considers both the additive‐genetic relationship, that is, the pedigree of individuals, and shared linkage groups, which link the tested Single Nucleotide Polymorphism (SNP) loci to causative loci (Habier *et al*., 2013). As a consequence, a more diverse population may be a more difficult target for genomic prediction, as more and smaller linkage groups have to be accounted for (Daetwyler *et al*., 2013).

In recent years, deep learning approaches for genomic predictions have gained attention (Ma *et al*., 2018; Montesinos‐Lopez *et al*., 2021). In contrast to conventional methods mentioned above, these do not use quantitative genetic models but are based on flexible arrangements of many non‐linear transformations of the input data (neurons) to detect (1) patterns in the input data, and (2) their relationship to the phenotype. The parameters of those transformations are optimized by supervised learning on a test set. This is expected to provide advantages where crop traits are strongly influenced by complex interaction effects that are not covered by the theory behind one of the more classical models (Pérez‐Enciso and Zingaretti, 2019). Besides this, neural networks training has linear time complexity with respect to sample size. This avoids the computing time explosion that researchers face whenever kinship matrices have to be inverted, like in the case of GBLUP (Pook *et al*., 2020). While in the beginning, multilayer perceptrons have been used for genomic prediction, recently convolutional networks have demonstrated their ability to capture linkage patterns (Pook *et al*., 2020). However, the full potential of deep learning has yet to be fully realized by providing much larger training sets than are available to single institutions. As such, a comprehensive evaluation of the advantages and limitations of deep learning techniques when applied to large data sets is urgently needed, particularly in the domain of plant breeding.

The prediction ability of genomic prediction, defined as the correlation between true and predicted phenotype, is significantly influenced by several characteristics of the training and test set, such as (1) the size of the population, (2) the diversity and relatedness between the genotypes, (3) the degree of linkage disequilibrium (LD) and (4) the quality of the phenotypic and genotypic data (Schopp *et al*., 2016). Increasing the training set is a straightforward and promising strategy to achieve high levels of prediction ability for diverse populations in hybrid wheat breeding practice (Zhao *et al*., 2021). Several steps in this direction have already been taken. For example, in a study including more than 8000 wheat landraces, prediction abilities of 0.68 for thousand kernel weight within the population could be achieved (Crossa *et al*., 2016). In another study, a massive data set of more than 10 000 wheat lines was phenotyped in an unreplicated single‐year design, and prediction abilities close to one for grain yield could be attained in a cross‐validation (Norman *et al*., 2018). These results are very encouraging but require either a large investment of resources beyond the reach of most institutes and companies or reduced phenotyping intensities that are below the standards of commercial wheat breeding for variety development in terms of numbers of environments and replications.

Multiple institutional and/or across‐company collaborations for mutual benefit are an attractive concept to increase the populations for training genome‐wide prediction models but are hampered by heterogeneous and non‐orthogonal (unbalanced) data. Commercial breeding trials are unbalanced in that they screen a large number of genotypes and evaluate their phenotypes in only a small number of environments. Selected genotypes from the first breeding stages are then evaluated in more environments in the next season. Therefore, as the reliability of the estimate of a candidate's performance rises, the number of available candidates drops. Combining several of such trials would produce a data set that includes a large number of early‐stage genotypes, but also late‐stage data for a larger number of candidates than what is feasible for each individual actor. In an earlier study, on which we build here, combining multiple historic wheat trials doubled the prediction ability for grain yield for hybrids (Zhao *et al*., 2021). Combining different data sets is therefore promising. In this study, we investigated the impact on genomic prediction of combining such historical data with routine breeding data from four companies. Our objectives were (1) to investigate whether it is possible to perform an integrated analysis of disparate phenotypic and genotypic data sets and how to perform quality control of such a task, (2) to examine what prediction abilities can be expected when using genomic prediction beyond the confines of individual experimental series and how well multiple series can be combined to form larger training sets for genomic prediction as well as to explore the potential of deep learning models for enhancing this process and (3) to test approaches to improve the training set by drawing subsets from the full data, distilling the most reliable data and potentially increasing prediction ability.

## Results

### Absence of genetically divergent subpopulations revealed by accurately imputed genotypic data

**Figure 1:**
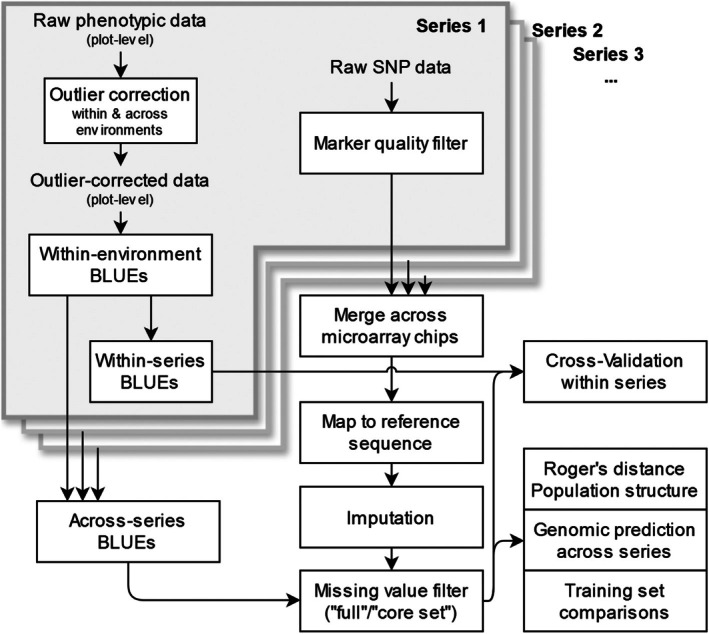
Schematic overview of the data‐processing steps.

**Figure 2:**
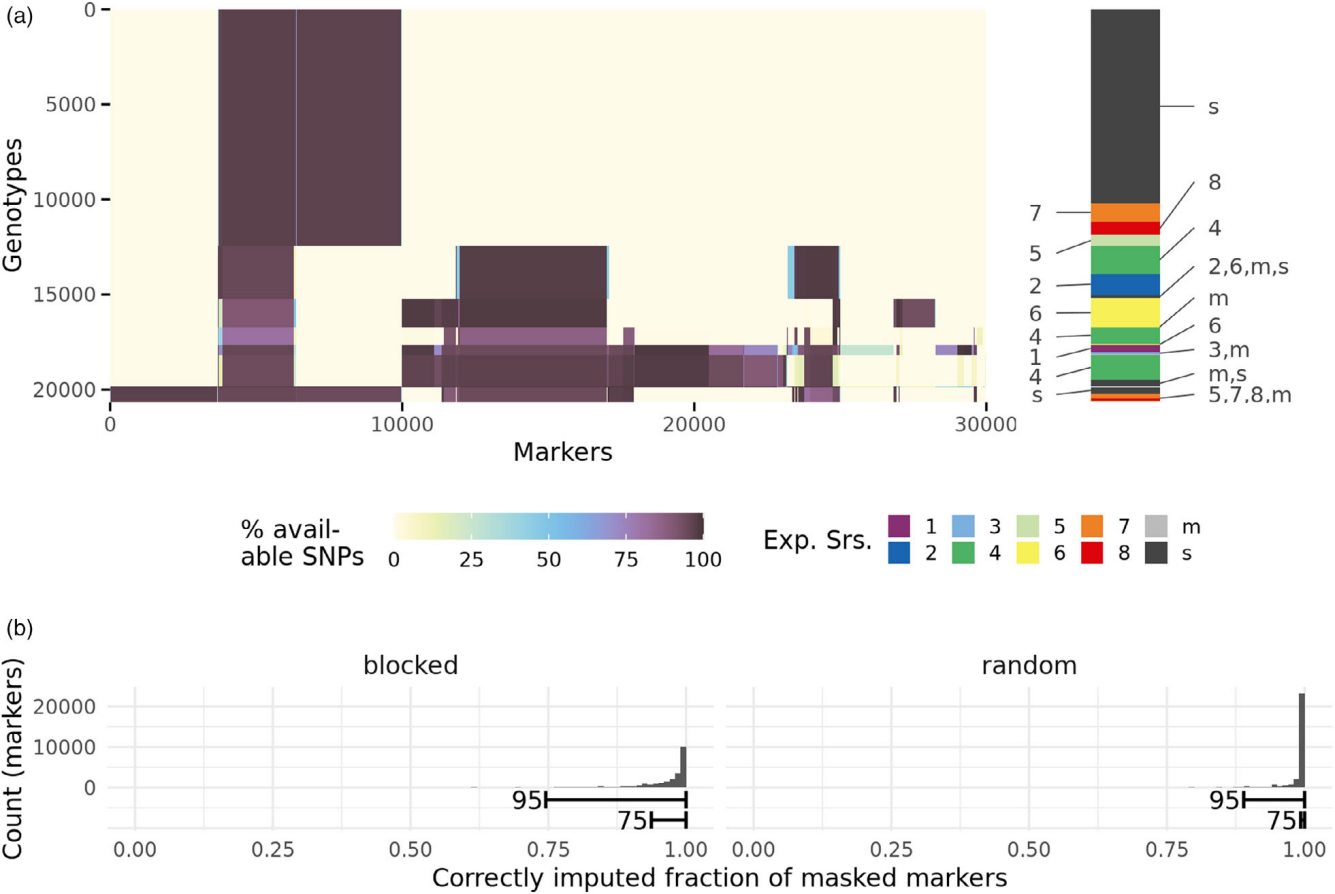
Imputation data basis and accuracy. (a) Available single‐nucleotide polymorphism (SNP) calls for different experimental series after quality filtering and merging of individual genotyping batches and before imputation. Dark areas indicate available genomic data for the respective markers and genotypes. Both are ordered to cluster markers/genotypes with similar availability together. The experimental series that genotypes belong to is indicated on the right side by colours and labels. Labels of multiple adjacent small groups are merged to reduce visual cluttering. The digits indicate the experimental series, ‘s’ indicates genotypes that have no phenotypic data (only genomic data), and ‘m’ indicates genotypes that appear in multiple experimental series, for example, common reference genotypes. (b) Imputation performance estimated by partially masking and imputing known genomic data. Histograms show the fractions of correctly imputed calls per marker, so the baseline for random guessing is 0.5. The markings ‘95’ and ‘75’ encompass the respective percentiles: 95% and 75% quantiles, respectively. The two subplots ‘blocked’ and ‘random’ discern the two different masking strategies (mask many SNP calls for single genotypes or randomly mask SNP calls, see **‘** Methods’).

Given the block‐wise gaps in the SNP data resulting from the integration of the heterogeneous SNP array platforms (Figures 1 and 2a), we conducted a validation of the imputation accuracy. This was estimated by masking and imputing some SNP data in a blocked and a random approach and then calculating the ratio of correctly imputed SNP calls to the masked SNP calls. In the blocked approach, almost all markers were imputed with accuracies above 0.75 (95th percentile) and most (75th percentile) even above 0.93 (Figure 2b). Imputation using the random masking approach was possible with even higher accuracy. The 95% and 75%‐percentiles of the imputation accuracy were 0.89 and 0.99, respectively.

**Figure 3:**
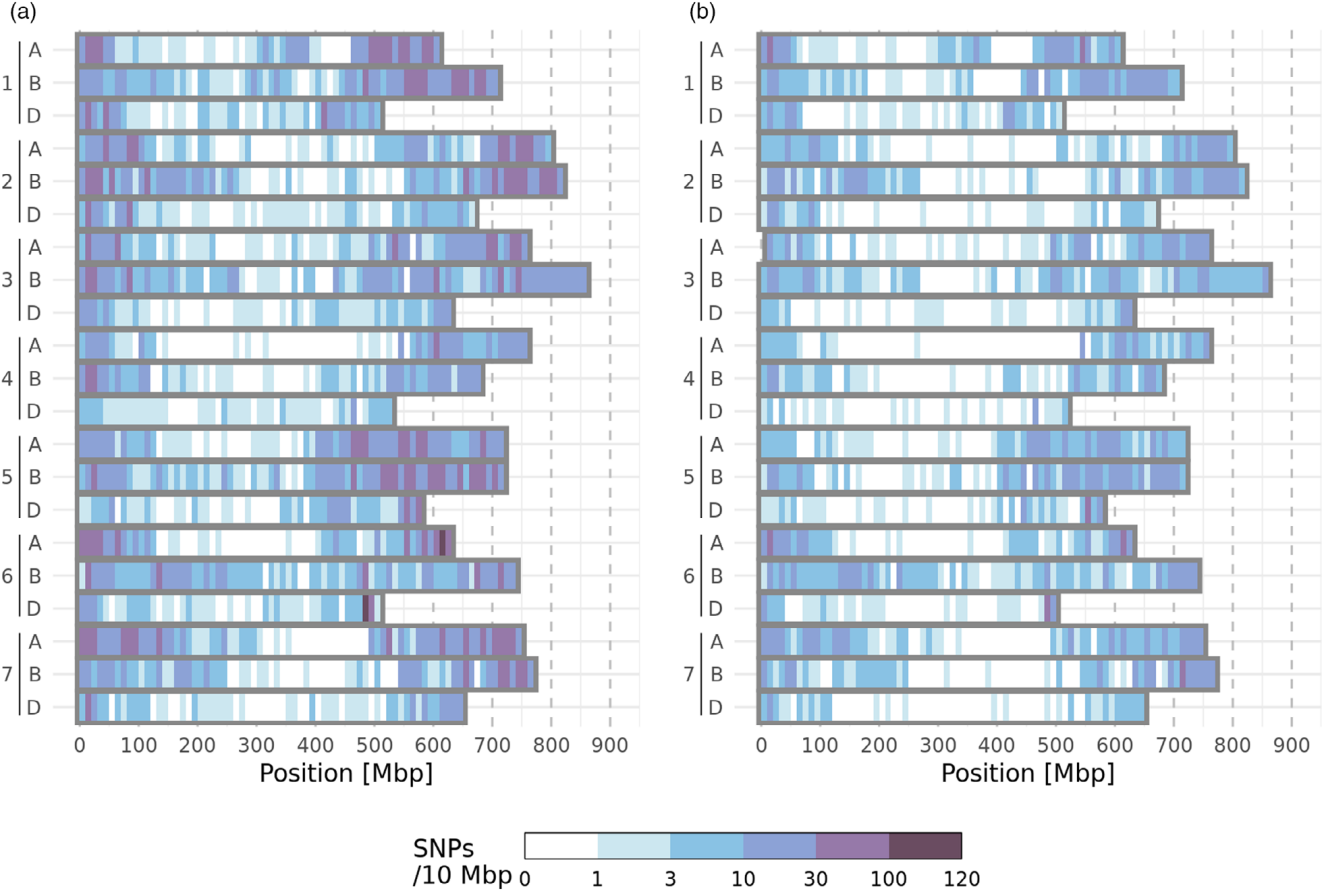
Density of SNPs used for genomic prediction when ordered according to their genomic position. Chromosome names are denoted on the vertical axes, nucleotide position on the horizontal axis. Density is shown by colour for 10 Mbp chunks. (a) Markers passing the liberal missing value threshold (<80%, 13 692 markers). (b) Markers passing the strict missing value threshold (<30%, 5913 markers).

The imputed SNP data covered most of the wheat genome (Figure 3a). Markers satisfying the liberal missing value criterion (at most 80% missing and imputed values) were found at densities of about 1–100 markers per 10 Mbp (Figure 3a). The smaller marker set resulting from the strict missing value criterion, that is, with at most 30% missing and imputed values, covered the genome at 1–30 markers per Mbp (Figure 3b). Coverage near the chromosome centres was markedly weaker than near the ends, and especially for the strict missing value criterion, large gaps were present near the chromosome centres.

**Figure 4:**
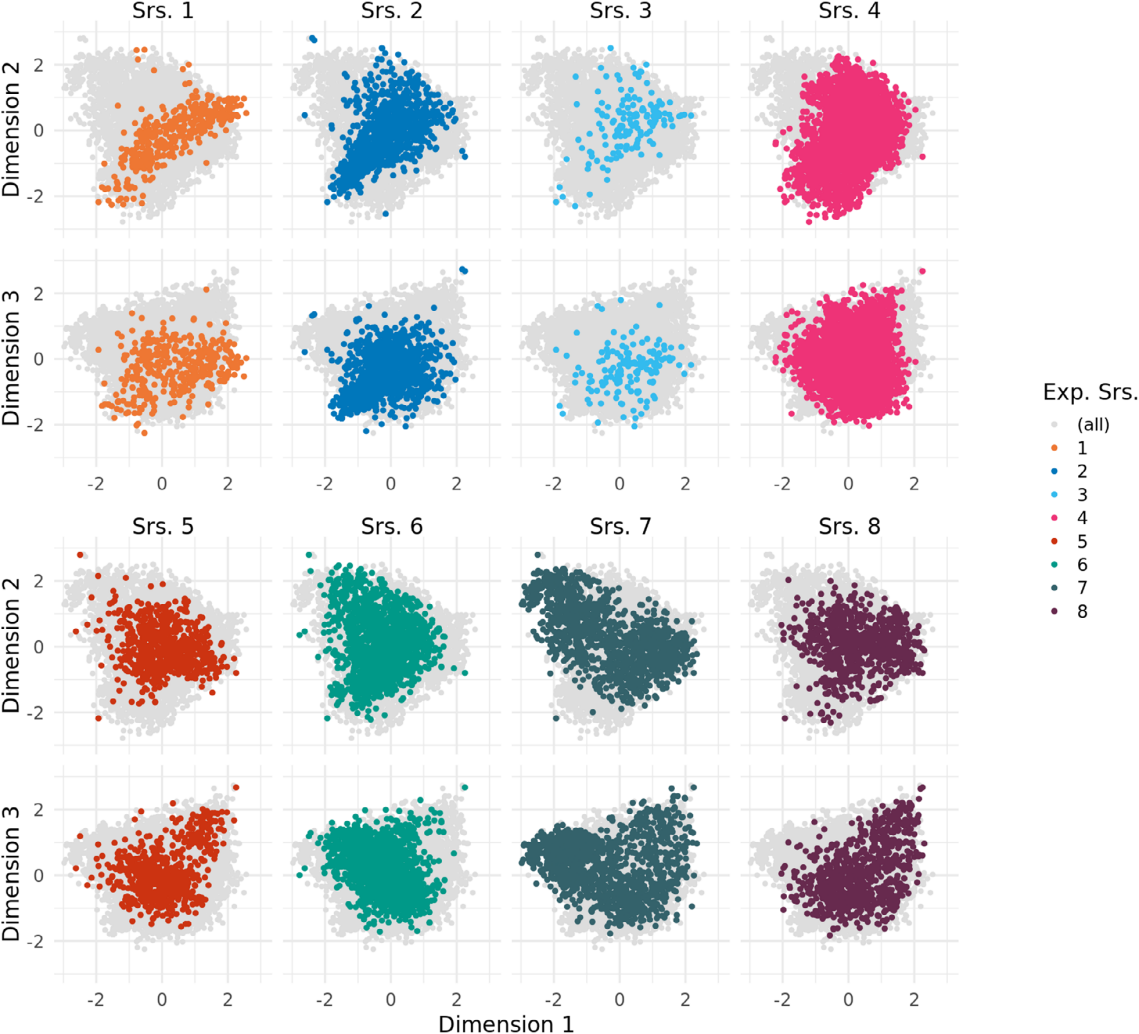
First three principal coordinates of the experimental series (Srs.), based on the Rogers' distances between lines.

The principal coordinate analysis based on the Rogers' distances revealed added diversity by combining the series of the study (Figure 4). Parts of some series were found in regions of the diversity space that were only sparsely covered by other series. For example, parts of series 6 and 7 were outside of the diversity space of series 1–3 but were quite similar to each other. Besides this tendency towards complementarity, none of the series formed a clearly distinct cluster separated from all other series.

### Phenotypic data are of high quality and consistent with genotypic data

**Figure 5:**
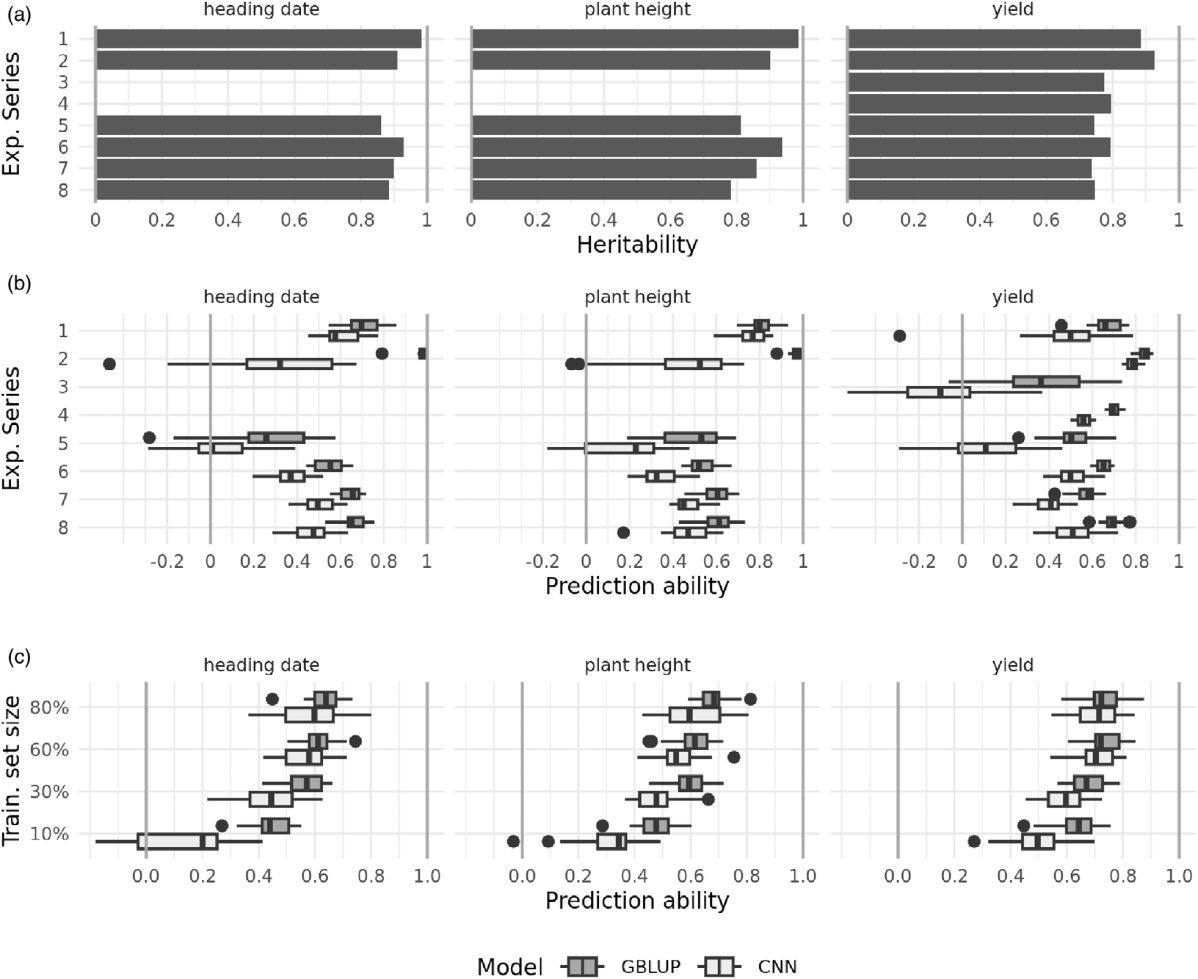
(a) Heritabilities of the individual experimental series. (b) Prediction abilities within each experimental series, 20 cross‐validation replications. (c) Prediction abilities across experimental series, 20 cross‐validation replications. Training set sizes are percentages of the full number of available genotypes for the respective trait (see Table 2). Cross‐validations in subfigures b and c were performed with both GBLUP and a CNN (boxplot colours).

We have integrated and curated phenotypic data generated in 105 000 grain yield plots as part of large public–private partnerships or wheat breeding programs in Central Europe. As a measure of the quality of the phenotypic data, broad‐sense heritability was estimated for three traits for each experimental series (Figure 5a). Heritabilities ranging from 0.86 to almost 0.98 were obtained for heading date, from 0.81 to 0.99 for plant height, and from 0.74 to 0.93 for grain yield. Thus, the quality of the phenotypic data was excellent for all traits.

Within‐series cross‐validation showed moderate to high prediction abilities. Medians across the 20 replications ranged from 0.50 to 0.98, except for series 5 for heading date (0.26) and series 3 for grain yield (0.36, Figure 5b). The latter can be explained by the fact that series 3 consists of only 142 inbred lines (Table 2). For series 5–8, which include data from commercial wheat breeding programs, the prediction abilities for heading date and plant height were lower than for the historical series. This can be explained by the unbalanced nature of these series. Due to economic efficiency constraints, these traits were evaluated in fewer environments than grain yield; in some cases, the average number of environments per genotype was even <2 (Table 1). Under these circumstances, an impact on the prediction ability is to be expected. Overall, the SNP data provided good predictions of the phenotypic data, indicating a successful data integration.

**Table 1:** Numbers of genotypes (gt), environments, that is, location times year combinations (env), per‐genotype average (avg) and total (tot), and number of tested years (y) by the experimental series. The second column shows the calendar year ranges. For series that contain both hybrids (h.) and inbred lines (i.) those two groups are shown on distinct rows

Series	Years (20)	Heading date				Plant height				Grain yield			
		gt	env avg	env tot	y	gt	env avg	env tot	y	gt	env avg	env tot	y
1	09–10	380	8.0	8	2	380	8.0	8	2	380	8.0	8	2
2 (h.)	16–19	3639	17.6	38	4	3639	18.3	39	4	5051	10.1	61	4
2 (i.)	16–19	469	18.5	38	4	469	19.6	39	4	1099	9.6	61	4
3 (h.)	12–13									1604	11.0	11	2
3 (i.)	12–13									144	11.0	11	2
4	12–15									4958	3.9	30	4
5	20–21	781	1.3	7	1	1001	2.1	8	1	1911	4.4	26	2
6	20–21	1631	5.3	13	2	1631	5.6	15	2	1631	5.7	17	2
7	20–21	1707	4.1	9	2	1742	4.0	10	2	1742	4.4	12	2
8	20–21	3516	1.4	7	2	3512	1.6	7	2	3505	2.1	15	2
(all)	09–21	12 096	8.1	81	8	12 347	8.3	83	8	21 891	6.2	168	12

### Convolutional neural networks become competitive with larger training set sizes

Training set sizes greatly impacted performance achieved with convolutional neural networks (CNN). For small training set sizes of 10% (for yield: around 950 genotypes, Table 2), the CNN showed lower prediction abilities by a margin of about 0.15 compared to GBLUP (difference of the medians, Figure 5c). Interestingly, as the training set sizes increased to 80% (about 7.600 genotypes for yield), this margin narrowed and CNN performed similarly to GBLUP, even exceeding it at a few individual iterations (Figure 5c). The rate at which this gap closed varied with the trait and for yield, where the largest training sets were available, the gap closed earlier than others.

**Table 2:** Effective population sizes ($N_{e}$) and numbers of genotypes (inbred lines only) available for prediction with both phenotypic and SNP data available for each experimental series

Series	$N_{e}$	Number of genotypes			
		Heading date	Plant height	Grain yield	(all)
1	34.5	371	371	371	371
2	51.6	467	467	1081	1081
3	49.1	0	0	142	142
4	58.0	0	0	3703	3703
5	61.9	214	418	641	641
6	55.0	1614	1614	1614	1614
7	29.7	1178	1213	1213	1213
8	40.7	848	848	848	848
(all)	79.4	4665	4904	9480	9480

We also benchmarked the performance of the CNN against GBLUP, with the data siloed into the individual experimental series. The training set sizes for these predictions were 90% of the genotype counts of the individual experimental series, which were 128 to 3332 genotypes in the example of yield (Table 2). High GBLUP prediction abilities for series were associated with a strong CNN performance, leaving only a small difference between CNN and GBLUP prediction abilities (Figure 5b): For example, for yield, the series 2 and 4 lead to the most accurate GBLUP predictions (0.85 and 0.69) and also the smallest gap (0.06 and 0.13) between GBLUP and CNN predictions. The picture was reversed for series 3, with both the worst median GBLUP prediction ability (0.36) and the largest gap to the median CNN prediction ability (0.46). The only outlier to this pattern is the experimental series 2 for heading date and plant height, where a high GBLUP prediction ability coincides with a large gap to the CNN performance.

We did not investigate the performance of the CNN in the remainder of the study but used GBLUP because of the high computational burden and the comparable or superior performance of GBLUP.

### Experimental series can be flexibly combined in genomic prediction training sets, approaching a plateau in their prediction ability

**Figure 6:**
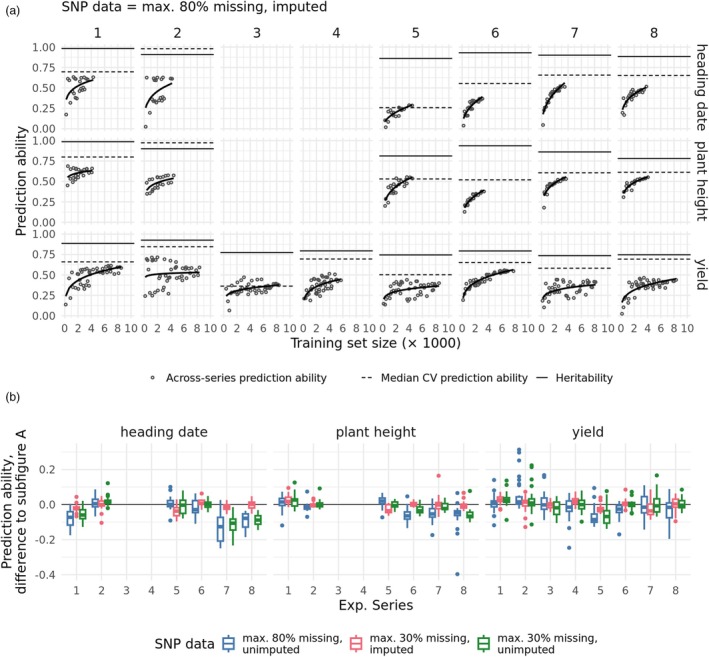
Across‐experimental‐series prediction abilities. (a) Prediction abilities using the large and imputed marker set (see ‘Methods’) and different combinations of experimental series as training sets. The training sets are ordered by their size (number of training genotypes) on the horizontal axis and by their prediction ability on the vertical axis. Each plot shows one experimental series as the test set and one trait. Heritability and median cross‐validation (CV) prediction ability (see Figure 5a,b) are annotated as horizontal lines for reference. The empirical model fit (Equation 5) is denoted by a solid line. (b) Difference in prediction ability when using any other genomic data than the large and imputed data. Each boxplot summarizes genomic prediction runs of the same test set and different series combinations as the training set. The colours show the results for different missing value thresholds and imputation of the SNP data (colours). The difference to the prediction abilities of subfigure a is shown.

To test the ability of predicting unknown genotypes given the collection of series in this study, we chose different combinations of the series as training sets and assigned all other series as test sets. We then derived the genomic prediction ability by comparing the predictions with the across‐series BLUEs of the test sets, calculating the prediction abilities separately for each series in the test set. Cumulating different combinations and numbers of series resulted in training sets of different sizes, by which the prediction abilities were ordered (Figure 6a). As a general trend, we observed an increase in prediction ability with an increasing training set size, but this increase approached a plateau beyond training set sizes of about 4000 individuals. For many test sets, the prediction abilities approached those obtained by using the series' own data in the cross‐validated genomic prediction (Figure 6a, dashed lines) but did not come close to the upper limit defined by the square root of the heritabilities (solid lines).

The above scenarios are based on imputed marker data and the liberal SNP filtering criterion (<80% of pre‐imputation missing values). Consistent with the high imputation accuracy of the missing SNP data, we also observed a decrease in prediction ability in genome‐wide prediction on average across the three traits when the same liberal filtering criterion was used but no missing values were imputed, or when the strict SNP filtering criterion (<30% of pre‐imputation missing values) was used (Figure 6b). Thus, liberal filtering combined with imputation also appears to be the most successful strategy for the block‐wise missing marker data underlying the used data set.

We then investigated whether the number of series included in the training set had a significant effect on prediction ability, given an unchanged training set size. We generated training sets of 800 genotypes coming from either single or multiple experimental series and compared the resulting prediction abilities: On average, using a single series resulted in a lower prediction ability of 0.02 for heading date and below 0.01 for plant height and grain yield (Figure S1). The standard deviations between replications were much larger, ranging from 0.07 to 0.08. The differences in standard deviation were below ±0.01 for all traits. Thus, for a medium‐sized training set, the number of experimental series included in the training set had no meaningful effect on the prediction ability.

### Most experimental series are compatible with each other for prediction

Observing the increase in genomic prediction ability with increasing training set size, we investigated whether the inclusion of individual series in the training set benefits the prediction ability for all or some test sets. As a starting point for this analysis, we derived the average increase of prediction ability with training set size using an empirical model (Equation 5, Figure 6a). We considered the residuals, which can be interpreted as the performance of the genomic prediction runs, corrected for training set size. We noticed that these deviations were small compared to the effect of the training set size, with a standard deviation of 0.09 for grain yield and heading date, and 0.05 for plant height. This can also be seen visually, with the genomic prediction abilities gathering closely around a common tendency for most test sets (Figure 6a).

We then decomposed the deviations of the genomic prediction runs into contributions of individual series in their training sets (6) to find out whether certain experimental series caused prediction runs to systematically over‐ or underperform. The influence of the choice of series corrected for training set size was small compared to the importance of increasing the training set size (Figure S2b). The total variance attributable to individual series or their combinations was higher for heading date (0.009) than for plant height and grain yield (0.003). Judging from the relative sizes of the variance components, the interaction effects of specific pairs of experimental series (one being part of the training set, the other being the test set) were the dominating factor. Main effects of individual series being in the training set, representing experimental series that improved or decreased prediction ability for most test sets, played only a minor role (Figure S2b). Only a few individual training set—test set combinations were unusually beneficial or detrimental for prediction ability. The series 1 and 2 showed particularly high compatibility, improving prediction ability by 0.1–0.3 above the values expected for the training set size for all three studied traits (Figure S2a). Apart from this, the combinations with the strongest deviations from the expectation showed no particular pattern (Figure S2a), for example, series 5 → 7 (training set → test set), with a prediction ability of −0.09 compared to the expectation, or series 7 → 8 and vice versa for heading date (+0.07/+0.09).

### Assuring environmental diversity improves training set performance

**Figure 7:**
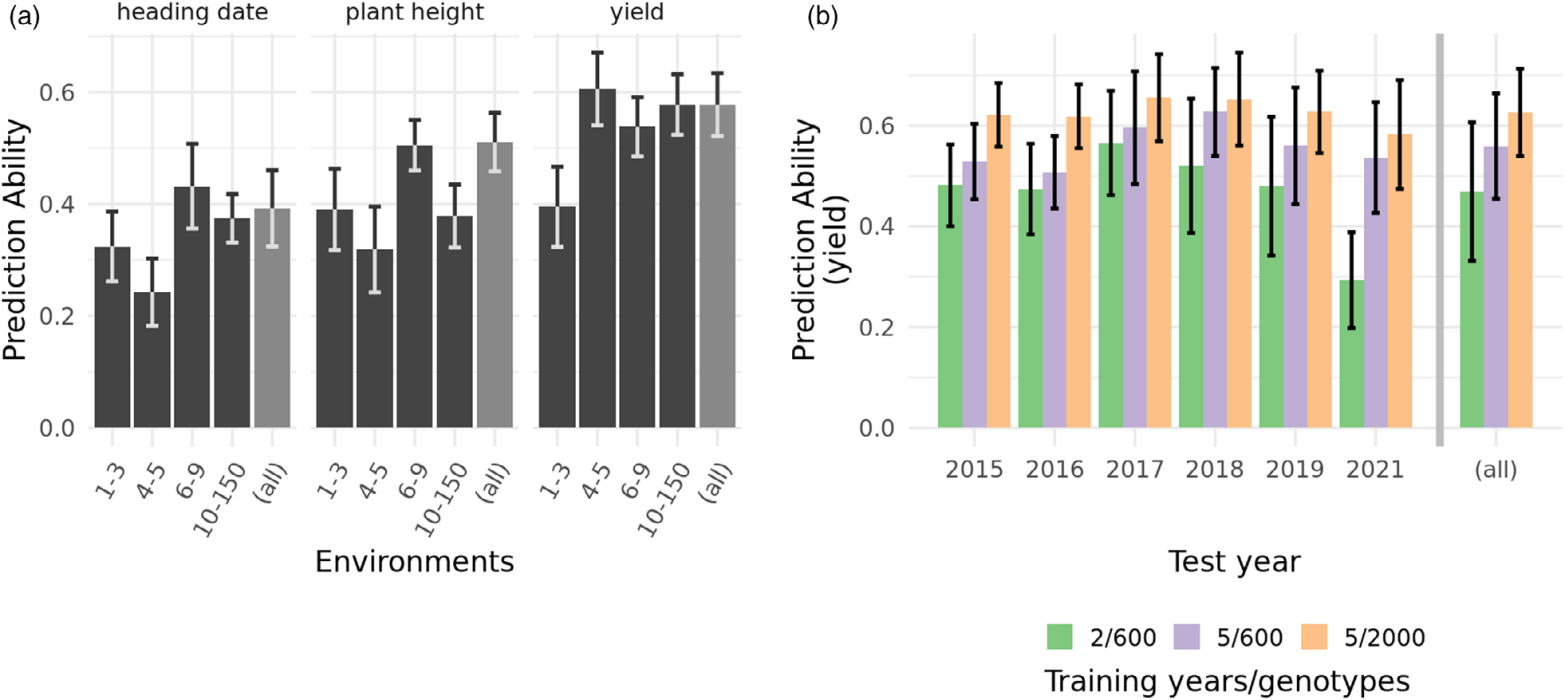
Prediction abilities for yield of across‐series training sets sharing breeding‐related characteristics. (a) Training sets of 300 genotypes each, formed of genotypes that were phenotyped in similar numbers of environments. For comparison, an equal number of training genotypes is drawn from all experimental series (‘(all)’). The test set was 100 genotypes, sampled to equal fractions from all experimental series. Error bars show the standard deviation of all replications (25). (b) Effect of using recent versus historical data for prediction. The test set was 100 genotypes drawn from a single year. Training sets were drawn from 2 to 5 years before the test set year. Additionally, results of a test set from the preceding 5 years and a higher number of genotypes are shown. Error bars show the standard deviation across the replications (50 per test set year). The label ‘(all)’ shows the mean and standard deviation of the pooled runs.

To test strategies that could further improve the performance of training sets for genomic predictions, we chose training sets encompassing defined numbers of environments or years, while keeping the training set sizes constant. The moderate training set sizes (300 and 600 for the first and second approach, respectively) resulted from restrictions from the unbalanced nature of the data. Following the first strategy, training sets backed by higher numbers of environments tended to yield better prediction accuracies (Figure 7a). However, compared to a training set of equal size sampled from the full data set without the environment number restrictions, these selections were not or only marginally better. For heading date and plant height, training sets backed by at least six environments per genotype were sufficient to reach the prediction ability of the random set. For grain yield, the prediction ability of the random set was reached with at least four environments per genotype.

A higher number of years in the training set was associated with a better prediction ability. For all years in the available data, including five preceding years in the training set proved superior to including 2 years in the training set at a constant training set size of 600 genotypes (Figure 7b).

## Discussion

### Sufficient interoperability to ensure successful integration of genotypic and phenotypic data

Previously, it has been shown that genotypic and phenotypic data from different public‐private partnership projects can be successfully integrated, doubling the prediction ability of hybrid performance in wheat (Zhao *et al*., 2021). In this study, we extended these efforts to data from inbred lines generated in four commercial breeding programs (series 5–8) and another published data set (series 1, Gogna *et al*., 2022). The first question to address is whether the new genotypic and phenotypic data are interoperable, which could be hampered either by differences of employed methods and protocols or by biological reasons.

The genotyping platforms employed are a significant factor influencing the interoperability of genomic data (Gogna *et al*., 2022; Schulthess *et al*., 2022a). In this study, all experimental series utilized SNP arrays, specifically the Illumina 90 k iSelect array (Wang *et al*., 2014). However, other genotyping platforms used in wheat research may have limited or partial overlap (Sun *et al*., 2020) and these platforms may be more suitable for characterizing populations with smaller linkage disequilibrium or distinct genetic substructures. As a result, ensuring interoperability of genotyping data across platforms may require additional efforts. Furthermore, the availability of marker sequences is critical for imputation of genomic data in our approach, although alternative methods exist that do not rely on marker sequences, albeit with a trade‐off in accuracy (He *et al*., 2015). Genotyping‐by‐Sequencing (GBS) technologies, which were not employed in this study, generate high‐density genotypic data and exhibit random patterns of missing values. These gaps can be imputed with higher accuracy compared to systematic gaps arising from the use of different marker platforms, as demonstrated in this and previous studies (He *et al*., 2015; Torkamaneh and Belzile, 2015). Therefore, we are optimistic about the integration of such data sources. When working with diverse landrace populations, where linkage disequilibrium is often reduced and/or genetically distinct subpopulations are present (Schulthess *et al*., 2022b), dedicated analytical approaches may be necessary to ensure the interoperability of genotyping data.

Interoperability of phenotypic data also represents challenges for data integration. There needs to be agreement on the definition of the recorded traits. In our case, we benefited from the fact that all data were focused on Germany, and thus the methods used in the German official variety tests (Test of Value for Cultivation and Use) served as a *de facto* standard of trait evaluation. Similarly, for the same reason, farming regimes in our study were similar, using intensive management practices to assess grain yield potential. To go beyond this scope, common method documentation standards need to be agreed, used and improved, balancing the fine line between covering many use cases and being understood and correctly employed by practitioners (Darnala *et al*., 2023; Papoutsoglou *et al*., 2020; Selby *et al*., 2019). Even then, objective phenotyping can be a challenging task in a commercial breeding program considering the trial network size, speed of plant development and restricted staff size.

In order to enable interoperability between series, common genotypes are essential to estimate environmental effects. The experimental series of this study were mostly connected by more than five genotypes, which is a standard also used in commercial plant breeding. Some combinations of series fall below this standard (Table S1), particularly for the historical series with the traits heading date and plant height. Fortunately, the connectivity of data sets can also be transitory, so that one experimental series which is well connected to two other series can serve as a common reference, relating them to each other even when they are mutually only weakly connected. Declaring genotypes with a Rogers' distance below a certain threshold as equal (Zhao *et al*., 2021) can further increase these numbers slightly (by on average three individuals in our case) for pairwise comparisons.

### Potentials and limitations of genome‐wide prediction across series

For predicting hybrid wheat performance, it was noticed that across‐population prediction profits from combining multiple different experimental series into one training set (Zhao *et al*., 2021): Prediction abilities of up to 0.4 were achieved. Here, we focused on inbred lines and drastically increased the number of available genotypes by cooperating with commercial breeders. We confirmed also for inbred lines that cross‐population prediction profits from combining multiple different experimental series into one training set and were positively surprised by the flexibility with which the different experimental series could be combined. The increases in prediction ability were largely determined by the size of the training set (Figure 6a), but which series were included in the training set was less important (Figure S2b). An important factor that contributes to this is the weak population structure in our joint population (Figure 4). Other studies, in particular those focusing on gene bank material like landraces, have found populations that form more distinct clusters (Crossa *et al*., 2016; Ramstein and Casler, 2019; Schulthess *et al*., 2022a). As the relatedness between the genotypes in the test and the training set drops, the prediction ability of GBLUP drops as well (Alemu *et al*., 2023; Habier *et al*., 2010; Lorenz and Smith, 2015). Therefore, when populations are found to diverge in their genetic diversity, the power of across‐population predictions will decrease. However, Central European elite wheat breeding pools seem to share large parts of genetic diversity. We conclude that in such populations, combining data from multiple breeding programs is a very promising strategy to improve genomic prediction ability and shows a way for small to medium‐size breeding programs to achieve shared benefits through cooperation. Experimental series 2 partly contains genetic resources from the IPK gene bank, but the PCoA (Figure 4) does not show a markedly larger genetic space covered by this series than by the other. Reasons could be that this diversity, weighted by the number of genotypes, is less prominent compared to the distances between the individual data sets and thus is not shown by the PCoA. Another reason could be the marker filtering before the Roger's distance was computed, which might have removed rare alleles that contributed to that population's diversity. However, within‐ and across‐prediction abilities are not lower for this series (Figures 5b and 6a), indicating that there is no strong effect of the removed markers on the measured traits. Traits like disease resistances, which can show higher dependence on individual loci, would require a more careful approach in this respect.

### Opportunities to further boost the prediction abilities

Expanding the training set beyond 4000 lines yielded diminishing returns in prediction ability, with performance approaching a plateau. However, it is worth noting that this plateau was reached well below the heritability of the test set, which represents the theoretical maximum (Figure 6a). The reasons for this are not clear yet and warrant further studies. First, when moving from within‐series to across‐series prediction, one loses the additional prediction ability which GBLUP confers by capturing co‐segregation when training and test genotypes are only a few generations apart (Habier *et al*., 2013). Another possible cause could be that the increased diversity of the joint data weakens the linkage between causative loci and the loci in the SNP data (Meuwissen, 2009). In a previous study, in order to quantify this effect in an applied setting, subsets of different nominal and effective population sizes were drawn from experimental series 4 and cross‐validated prediction accuracies, that is, prediction abilities divided by the square root of heritability, were obtained (Zhao *et al*., 2021). Extrapolating this relationship to the nominal and effective population sizes that were seen in our study for the joint population (Table 2), an expected prediction accuracy of about 0.75 would result. This would account for a large part of the missing prediction accuracies of the training sets in this study (Figure S3). According to this theory, increasing the ratio of the nominal to the effective population size should improve prediction ability. This could be done by selecting subsets for the full training data that are highly related to the test population. However, prior attempts to do this have rarely succeeded in surpassing the prediction ability of GBLUP using the full training data and mostly focused on achieving comparable power with a smaller training set size (Fernández‐González *et al*., 2024; Isidro y. Sánchez and Akdemir, 2021; Lopez‐Cruz and de los Campos, 2021).

A further avenue might be to test alternative genomic prediction methods; for example, BayesB has been reported to perform slightly better than GBLUP in estimating marker effects based on LD to causative loci (Habier *et al*., 2010). In recent times, attempts to use non‐linear machine learning methods, such as Random Forest, Support Vector Machine or Neural Networks to predict genotype performance, have reached parity with GBLUP and sometimes even achieve better results (Abdollahi‐Arpanahi *et al*., 2020; Montesinos‐López *et al*., 2024; Sandhu *et al*., 2021). To connect to these findings, we have employed a Convolutional Neural Network (CNN) for genomic prediction and compared the results to the GBLUP. We found that for our task, the CNN was able to predict phenotypes equally well as the GBLUP, where GBLUP provides high prediction abilities. Subpar GBLUP prediction accuracies were associated with CNN performances that were not only weaker in absolute terms but also showed a wider performance gap to GBLUP. It seems that some factors that are detrimental for GBLUP affect CNN disproportionately stronger. The most likely factor is the training set size. For the across‐series predictions with small training sets (Figure 5c) and within‐series predictions of small experimental series (Figure 5b), poor CNN performance is to be expected. In order to fit a CNN, a high number of parameters and hyper‐parameters have to be estimated. On the contrary, for GBLUP, only a mean and a variance parameter have to be estimated, and predictions can then be derived by means of quantitative genetic theory. Therefore, small training sets are expected to be insufficient for an adequate training of a CNN, and consequently, GBLUP remains the preferred model for training set sizes smaller than about 4.000 genotypes. An interesting exception to this trend is experimental series 1, where both the CNN and the GBLUP attain high prediction accuracies despite the small training set size.

The observed comparable prediction performances of GBLUP and CNNs is consistent with examples from the literature (Montesinos‐López *et al*., 2024; Sandhu *et al*., 2021). However, those studies used only a fraction of the genotypes that were available for this study. Therefore, it stands to reason that there might be more factors than sample size that inhibit an even better prediction ability and eventual superiority of neural network‐based predictions over linear methods like GBLUP. Interestingly, there could be a counterpart for neural networks to the above‐mentioned hypothesis that the diminishing role of additive‐genetic relationships in multi‐origin data sets might hamper GBLUP predictions. The neural networks could strongly base its predictions on additive‐genetic relationship, neglecting the effects and interactions of individual alleles. This phenomenon of ‘shortcut learning’ was observed before for genomic prediction with neural networks (Ubbens *et al*., 2021). Neural networks have many properties that hold great promise for genomic prediction, like a linear time complexity with increasing sample size, their flexibility to incorporate many different data types like environmental covariates (Washburn *et al*., 2021), and their flexibility in cases where the additive genetic model falls short of describing the genetic architecture (Pérez‐Enciso and Zingaretti, 2019). The more interoperable training data can be made available, possibly by cooperation across institutional borders, the more of this potential can be realized. In this study, the IPK has served as an ‘academic data trustee’. By providing a neutral and confidential data deposit, commercial stakeholders could contribute data. The results suggest that such a model could facilitate innovation in breeding.

Besides the method, one could consider a more detailed genomic picture by increasing the marker density. In theory, the number of required markers for high genomic prediction abilities rises with the effective population size of the data set because the size of linkage blocks decreases (Meuwissen, 2009). However, the benefits of increasing marker density have already been found to diminish beyond about 5000 markers (Zhao *et al*., 2021), using experimental series 2–4 of this study. As the effective population size of this study remains at the same level, it seems unlikely that using more than the 13 000 markers used in this study would hold much potential for improvement. A limiting factor when comparing the effective population sizes of this study and that of Zhao *et al*. (2021) is that as hybrids are not used for genomic prediction in this study, the number of used markers differs and imputed genomic data plays a larger role in our study. Another approach to better model the correlation of genetic values for GBLUP would be to create trait‐specific correlation matrices. Employing the genomic relationship matrix to this end is a simplification which assumes that effective loci are very large in number and pervade the whole genome evenly. Instead, genetic correlation matrices could be computed, for example, from results of Genome‐Wide Association Studies (GWAS). To define the genetic correlation, only markers would be considered that are associated with the trait of interest, or by defining synthetic markers that are associated with specific haploblocks (Jiang *et al*., 2018; Weber *et al*., 2023).

Besides considering whether the training set data have sufficient power to capture the genetic architecture of the trait, one must also consider whether the genotypic values themselves are measured accurately enough. In particular, genotype‐times‐environment interactions could limit the prediction ability in this study. Where many environments are sampled, the proportion of genotype‐times‐environment interactions in the phenotypic variance of grain yield has been found to be large, sometimes even larger than the additive genetic effect (De Los Campos *et al*., 2020; Jarquín *et al*., 2014; Lado *et al*., 2016). When fewer environments are sampled, the impact of genotype‐times‐environment interactions is estimated to be less important (Montesinos‐López *et al*., 2024). Continuing this trend, where only one environment is sampled, and thus the experimental design confounds genotype‐times‐environment interactions with the genotypic main effect, GBLUP can achieve grain yield prediction accuracies of up to one (Norman *et al*., 2018). Interestingly, a larger number of sampled environments does not necessarily require a larger number of plots to be tested. The overlap between environments could also be improved by performing preliminary grain yield tests using more environments of a testing network, sparsely distributing candidates and thus keeping the overall number of plots constant. This has been shown to be advantageous in previous studies for hybrid series 2 and 3 (Lell *et al*., 2021; Zhao *et al*., 2021), as well as for biparental populations of barley and maize (Endelman *et al*., 2014). In the unbalanced breeding trials of this study, the largest share of the data is made up of early‐stage candidate varieties which are tested in less than five environments, and almost no series test genotypes in more than 10 environments per year (Table 1). Moreover, the number of years per tested genotype is small. Therefore, one can expect that the genotype‐times‐environment interactions are partially confounded with the genetic main effects in the across‐environment BLUEs. This assumption is supported by our observation that given an equal number of training genotypes, increasing the number of years in the training set improves the prediction results (Figure 7b). The across‐series predictions in this study use BLUEs of a single series in the test set. Therefore, it is conceivable that the across‐series genomic prediction will yield breeding value estimates that are in fact less affected by confounding genotype‐times‐environment interactions than the single‐series BLUEs to which they are compared. The confounding genotype‐times‐environment interactions in the test set BLUEs would therefore decrease the prediction ability. If this effect proves to be relevant, across‐series predictions would also be of value to breeders whose data sets exceed the aforementioned threshold of a training set size of about 4000 genotypes. Candidate varieties are tested in an extensive set of environments during official registration procedures, which exceeds the number of environments of the final breeding stage. Therefore, benchmarking genomic prediction methods using the final‐stage genotypes of one's own breeding program may give overly optimistic results due to residuals confounding genotype–times–environment interactions. Obtaining phenotypic and genomic data from official variety tests would therefore be a gold standard for assessing the impact of cross‐series predictions on prediction ability. This endeavour is easily hampered by legal issues, but seems worthwhile.

## Methods

### Plant material and field trials

Eight different experimental series were assembled for this study. The first four of them consist of historical data that have been studied previously. In addition, four more were contributed by breeding companies for this study. Within the experimental series, the number of genotypes ranges from 380 to 5051 and the number of environments (location – year combinations) from 8 to 61 (Table 1). The series are characterized in the following:

- Series 1: Orthogonal grain yield trials of 380 genotypes representing a broad diversity of the European elite breeding pool (Gogna *et al*., 2022).
- Series 2: Non‐orthogonal grain yield trials of 1099 diverse inbred lines including elite lines and plant genetic resources along with 5051 hybrids (Zhao *et al*., 2021, Experimental series II to V).
- Series 3: Orthogonal grain yield trials of 135 elite parent lines, their 1604 hybrids and 10 released varieties (Zhao *et al*., 2021, Experimental Series I, and Zhao *et al*., 2015).
- Series 4: Non‐orthogonal grain yield trials, generated in the course of a commercial inbred line breeding program, comprising 4958 genotypes (Zhao *et al*., 2021, Experimental Series VI).
- Series 5–8: Four experimental series, provided by four breeding companies. The series consisted of 781–3516 genotypes tested in an average of 1.3–6.7 locations in 2 years (Table 1). All four series were excerpts from the companies' usual breeding activities and were therefore non‐orthogonal grain yield trials in which lines were phenotyped and selected in up to 3 years. There are three sub‐trials per series, reflecting the breeding trial stages. The candidates were evaluated for grain yield under intensive treatment and partially for heading date and plant height. Connectivity between data from each series was ensured by several common genotypes (Table S1).

### Phenotypic data analysis

We applied a two‐step approach to analyse the phenotypic data as described previously (Lell *et al*., 2024) including outlier correction following the method M5 from Bernal‐Vasquez *et al*. (2016). See Figure 1 for an overview. Best linear unbiased estimator of the genotypes (BLUEs) within and across the environments was obtained after outlier correction. The within‐environment BLUEs were generated using the following mixed linear model:

(1) $$y_{\text{ijkl}}=\mu +g_{i}+t_{j}+r_{\mathrm{jk}}+b_{\mathrm{jkl}}+\varepsilon_{\text{ijkl}},$$

where y is the plot‐level grain yield data, μ is the overall mean, g is the genotypic effects, t, r and b are design effects for trials, replications and blocks, ε are residual effects, and i, j, k and l are indices for model effects. Depending on the experimental design, only some or none of the effects t, r or b were estimated. When phenotyping was done in an unreplicated trial in an environment, design effects were estimated and subtracted from the measurements. The design effects were estimated as random effects, *g* was estimated as a random effect to calculate BLUPs and estimate the genetic variance and as a fixed effect to obtain BLUEs. All random effects were identically and independently distributed. The repeatability was estimated as $r^{2}=\sigma_{g}^{2}/\left(\sigma_{g}^{2}+\sigma_{\varepsilon }^{2}/n\right)$, where $\sigma_{g}^{2}$ is the estimated genotypic variance, $\sigma_{\varepsilon }^{2}$ is the estimated residual variance, and n is the average number of plots per genotype. Environments that have a repeatability of <0.3, or that show <0.1 correlation to all other environments on average were discarded.

The across‐environment BLUEs were obtained using the following mixed model:

(2) $$\begin{array}{c} y_{\mathrm{ijk}}=\mu +s_{i}+g_{j}+e_{k}+\varepsilon_{\mathrm{ijk}}, \end{array}$$

where y are the within‐environment genotype means, μ is the overall mean, s are the experimental series effects, g are the genotypic effects, e are the environment (location × year) effects, ε are the residual effects, and i, j, and k are indices for model effects. The effects s are estimated as fixed effects, e are random effects, and g is fixed for BLUEs and random for BLUPs. The broad‐sense heritability was estimated as $H^{2}=\sigma_{g}^{2}/\left(\sigma_{g}^{2}+\sigma_{\varepsilon }^{2}/n\right)$, where n is the average number of environments a genotype is measured in, and $\sigma_{g}^{2}$ and $\sigma_{\varepsilon }^{2}$ are the estimated genetic and residual variances, respectively. All computations were performed using R 4.0.4 (R Core Team, 2021) linked to OpenBLAS 0.2.20 (https://www.openblas.net) and AsReml‐R 4.1.0.110 (Gilmour *et al*., 2015) on a Linux machine with 4 Intel Xeon CPU E7‐4890 v2 processors (120 logical cores) and required about 500 GB of RAM.

### Genotypic data analyses

All experimental series had SNP data available for varying fractions of genotypes (Table 2). The SNP data were generated according to each data provider's own processes and thus a heterogeneous selection of SNP markers was available for the different experimental series (Figure 2a). The SNP calls were filtered individually for each genotyping batch according to the following thresholds: Genotypes were discarded if they had more than 30% missing values or more than 10% heterozygous calls. Markers were discarded if they had more than 90% missing values or more than 20% heterozygous calls. As the SNP data were generated by different providers, we further checked and corrected for consistency of strand designation using overlapping genotypes before integrating data across experimental series. The computational efficiency of this process was improved by modelling SNP array overlaps as graphs and determining maximum spanning trees (Markowski *et al*., 2021). Marker positions were derived using BLAST (Camacho *et al*., 2009) and the Chinese Spring Reference Sequence v.2.1 (Zhu *et al*., 2021), removing markers whose sequences showed mismatches to the reference sequence or mapped to more than one position. Of the markers that showed variation for the population, a unique physical position was found for 29 970 markers and those marker's data were subsequently imputed. Imputation was performed using BEAGLE 5.2 (Browning *et al*., 2018) without the use of a reference panel for phasing information, with a window size of 1000, an overlap size of 100, and 10 burn‐in iterations.

As the SNP data are heterogeneous in terms of marker density, there are non‐random gaps in the SNP data, so that large numbers of markers are available for only a fraction of individuals. To assess the accuracy of imputing those systematic gaps, we chose a blocked masking approach: We divided the available markers into a low‐density set and a high‐density set by the fraction of genotypes that had data for the respective marker. Markers for which more than 70% of genotypes had data were grouped into a low‐density set; all other markers formed the high‐density set (low‐density set: 2582 markers, high‐density set: 13604 markers). For a random 10% of genotypes that had data for more than half of the high‐density markers, the high‐density marker data were masked. The resulting data set was imputed using the same BEAGLE parameters as described previously. For each marker, the imputation accuracy was calculated as the sum of masked SNP calls whose imputed data matched the original calls divided by the total number of masked SNP calls. This process was repeated 20 times. In an additional experiment, we employed a random masking approach to mimic missing calls as occurring, for example, in genotyping‐by‐sequencing approaches. We masked 1% of all marker calls randomly throughout the whole data set, imputed them, and calculated the imputation accuracy per marker as described above. This process was also repeated 20 times.

To visualize the population structure, we calculated the Rogers' distance (Rogers, 1972) using the SNP data set before imputation (Figure 2a). We filtered the data for a maximum missing value rate of 80% per marker and a minimum minor allele frequency of 0.05. This retained 13 720 markers of the initial data. Rogers' distances were computed for each pair of genotypes based on markers that were available for both. The Rogers' distances were then subjected to principal coordinate analysis using the R function ‘cmdscale’.

### Genomic predictions within and across experimental series

Within each experimental series, the correspondence of phenotypic and genotypic data was assessed. For this, a random sample of 90% of genotype means within each series were taken as training set to predict the remaining 10%. This process was repeated 20 times for each experimental series. To obtain the genotype means within each series, model (2) was fitted on the within‐environment BLUEs without the series effect s. The resulting genotypic effects (within‐series BLUEs) were then used as measurements in a genomic BLUP (GBLUP) model:

(3) $$\begin{array}{c} y_{i}=\mu +g_{i}+\varepsilon_{i}, \end{array}$$

where *y* were the within‐series BLUEs, μ is the mean, ε are the residual effects, and g are the genotypic effects that were modelled to be correlated using the VanRaden (2008) genomic relationship matrix K, so $\mathrm{Cov}\left(g\right)=K\sigma_{g}^{2}$, where the variance $\sigma_{g}^{2}$ was estimated by the model. The SNP data used to compute K were filtered to include only markers that have SNP calls for at least 80% of that series' genotypes and in addition a minor allele frequency of at least 0.05. The prediction was performed using the BGLR R package (Pérez and de los Campos, 2014).

We created many different training sets for genomic prediction by choosing different combinations of experimental series as training sets. From the large number of possible combinations, we chose a subset which was numerically optimized for D‐optimal experimental design using Fedorov's algorithm (Wheeler, 2004), with 135–349 different series combinations as training sets, depending on maximum training set size for the trait. We then determined the prediction ability of experimental series that were not included in the respective training sets.

To test the influence of quality‐control steps during data integration, we performed the predictions with four different versions of the SNP data, which influenced the genomic relationship matrix K: Markers were filtered using either a liberal threshold of at most 80% missing values per marker or a strict threshold of at most 30% missing values per marker. The data resulting from the liberal threshold had 13 692 markers. The data resulting from the strict threshold had 5913 markers. Furthermore, we used either the SNP data with or without imputation by BEAGLE. In the unimputed case, gaps in the marker data were filled by the respective marker means as described above. For the VanRaden distance, which involves centring the SNP data, this is equivalent to basing the pairwise distances only on markers for which there is information in both involved individuals. The resulting kinship matrices are used as covariance matrices for a genomic BLUP using the following model:

(4) $$\begin{array}{c} y_{i}=\mu +g_{i}+\varepsilon_{i}, \end{array}$$

where y, μ, g and ε were defined analogous to (3). Like for the within‐series cross‐validations, the computation was performed using the BGLR R package.

Subsequently, we focused on a more detailed analysis of the influence of individual series on the prediction ability. We based this on the prediction runs with the imputed SNP data with the liberal missing value threshold. For each test set i ($\;i\in \left\{1,\ldots ,d\right\}$, where d=8, the number of series in this study), a model

(5) $$\begin{array}{c} y_{\mathrm{ip}}=\alpha_{i}{x_{\mathrm{ip}}}^{\left(1/\beta_{i}\right)}+r_{\mathrm{ip}} \end{array}$$

was fitted to approximate an average increase in prediction ability with training set size, where $y_{\mathrm{ip}}$ was the prediction ability of the genomic prediction run of test set i that used the set of experimental series p as training set. $x_{\mathrm{ip}}$ was the number of genotypes in its training set, $\alpha_{i}$ and $\beta_{i}$ were empirical parameters, estimated separately for each test set, and $r_{\mathrm{ip}}$ was the deviation of the prediction run $\left(i,p\right)$ from the empirical average. Most of the genomic prediction runs had more than one series as training set, which is why i is an index and p is a set of indices.

We studied how individual experimental series being in the training set influence the prediction ability of a particular test set by fitting a linear mixed model on the vector r of deviations from the empirical fit (5) for all training and test sets. In the following, we denoted the deviation of a single genomic prediction run as $r_{\mathrm{ip}}$, indexed by the experimental series as test set i and the set p of experimental series indices in the training set. Based on this, the linear model was

(6) $$\begin{array}{c} r_{\mathrm{ip}}=\mu +\eta_{i}+\sum_{j}^{d}\delta_{j\in p}\left(\theta_{j}+\kappa_{\mathrm{ij}}\right)+\varepsilon_{\mathrm{ip}}\\ \;\text{with}\;\delta_{x}=\left(1\;\text{if}\;x\;\text{is true},\text{else}\;0\right). \end{array}$$

The model decomposes the deviations of the genomic prediction runs into a mean μ and three groups of random effects: The main effects of the test sets (η) and training sets (θ) with d effects each, and the effect of each combination effects κ of two experimental series as test set i and training set j. For each of η, θ and κ, one variance parameter is estimated. As one run has more than one training set, the term $\delta_{j\in p}$ selects for each measurement $r_{\mathrm{ip}}$ the relevant parameters. The residuals are denoted as ε. The model was fitted using the BGLR R package as Bayesian Ridge Regression (model setting ‘BRR’).

### Convolutional neural network for genomic prediction

To compare with GBLUP, we used a Convolutional Neural Network (CNN) to assess the prediction ability of both within‐series BLUEs (previous section) and across‐series BLUEs. The Python framework ‘Keras’ was used for model development. The CNN operated on a one‐dimensional sequence of marker states, ordered by their mapping position in the genome. The two homozygous states were coded as 0 and 2, and the heterozygous state as 1. Both phenotypic and marker state vectors were rescaled to a range of [0, 1].

For the training set, we selected random samples of 10%, 30%, 60% and 80% of the total data available for each trait. The test set consisted of 100 genotypes that were not in the training set, randomly chosen for each iteration. The process was repeated for 20 runs for each trait and training set size. The CNN was designed to allow a flexible network architecture for each run, with neurons as edges in an acyclic directed graph, organized into several layers to capture linkage and haplotype structure, and genetic interactions, respectively.

The first set of layers focused on feature extraction, followed by layers for pattern recognition. The specific network structure was influenced by a variety of hyperparameters, whose values were optimized using the Hyperband Tuner (Li *et al*., 2017). The hyperparameter space for the feature extraction section included (1) number of alternating convolution and average pooling layers (three to five), (2) number of filters (ranging from 64 to 512 with a step size of 64) and (3) kernel size (between three and 36 with a step size of three) in the convolution layer. The pool size for the pooling layer was also varied between two and 32 with a step size of 4. The feature extraction output was flattened and relayed into the pattern recognition section.

The hyperparameter space for the pattern recognition section comprised (1) the number of alternating dense and dropout layers (one to four), (2) the number of dense layer units (between 32 and 256 with a step size of 32) and (3) a dropout rate (between 0.1 and 0.5 with a step size of 0.01). Dropout was applied to reduce overfitting by preventing the model from becoming too reliant on specific neurons. The Rectified Linear Unit (ReLU) function was used as the activation function for all layers except the final prediction neuron. A single neuron with a hyperbolic tangent (tanh) activation function performed trait prediction, receiving input from all neurons in the final pattern recognition layer.

For each set of hyperparameters chosen by the tuner, the model was fitted with a batch size of 32 genotypes. The training set was split into 90% to be used for this purpose and 10% that were used exclusively to compare the prediction performances of the trained models resulting from the hyperparameter choices (validation set). The goal of the tuner was to minimize the mean squared error between predicted and observed genotype means in the validation set. Hyperparameter tuning stopped when the error did not decrease by more than 0.01 over 5 or more iterations. The resulting model was used for genomic prediction.

For comparison with GBLUP, the same across‐series data as used for the Neural Network was subjected to GBLUP following Equation (4).

### Influence of number of experimental series, environments and years in training set

We measured the effect of including a single versus multiple experimental series into the training set, at a constant number of 800 genotypes in the training set and 100 genotypes in the test set. Both sets were chosen randomly (25 replications). In the first scenario, we choose all training genotypes from one random experimental series only. Only the experimental series 6, 7 and 8 for heading date and plant height and series 2, 4, 6, 7 and 8 for grain yield were large enough for this. In the second scenario, we sampled the same number of genotypes randomly from all but one series. The test set was sampled from the series that were not in the training set.

We further investigated potential strategies to subset genotypes based on data quality or number of phenotyping environments to improve the predictions obtained from a large integrated data set by restricting the training set to fulfil different criteria. To test the influence on prediction ability of the number of environments a training set is based on, we assigned all genotypes to one of four environment groups: 1–3, 4–5, 6–9 and 10 or more environments. These were chosen based on the available data to have groups as even in size as possible while still including genotypes from multiple experimental series into one environment group. The training set consisted of 300 genotypes randomly chosen from the genotypes of an environment group. The test set were 100 genotypes that were randomly drawn while ensuring that genotypes from each experimental series were included at equal shares in the test set. For each environment group, 25 replications of the prediction were performed. For the prediction, the GBLUP model (3) was used, and the kinship matrix was based on the imputed SNP data using the liberal missing value criterion (13 692 markers).

To test whether historic data were still useful for predictions, we selected training sets that had the same number of genotypes but differed in the number of years covered by the training data. We chose 2‐year ranges from which we sampled 600 training genotypes for each range: 1–2 years before the test set and 1–5 years before the year of the test set. Our data covered 12 different years, so we could generate test sets for 7 years (year 6 to year 12). Analyses was only done for grain yield because the available data for heading date and plant height was missing for several environments. We also excluded one test set (2020) because the preceding years did not allow for a training set of adequate size. The test set encompassed 100 random genotypes that were measured in the respective year. As mentioned before, we used the same set of pre‐computed across‐environment BLUEs as data for the predictions, so technically information from more than 1 year was included in some of these BLUEs. However, the majority of genotypes in our data were measured in 1 year only. We made sure that genotypes that were included in the training set were not included in the test set. The prediction was done using the GBLUP model (3) and the kinship matrix was based on the imputed large SNP data.

## Author Contributions

M.L., Y.Z. and J.C.R. conceived and designed the study. U.A., J.D., W.M.E., T.E., M.G., M.Ki., M.Ko., S.K., N.P., M.R., V.W. and M.W. acquired, curated and contributed data. M.L. curated and processed the data, performed the analyses and analysed the results. Y.Z. supported data analyses. A.G. conceptualized the neural network. M.L., V.K. and A.G. performed neural network‐based data analysis. M.L., Y.Z. and J.C.R. interpreted the results and wrote the manuscript, with the help of all coauthors. Since the study was conducted, Valentin Wimmer has moved to Aardevo B.V., Johannes Postweg 8, 8308 PB Nagele, Netherlands, and Martin Kirchhoff has moved to Nordzucker AG, Küchenstraße 9, 38 100 Braunschweig, Germany.

## Competing interests

The authors declare that they have no competing interests.

## Supporting information


**Table S1** Number of overlapping genotypes between experimental series.
**Figure S1** Comparing prediction abilities using only one (‘single’) versus multiple (‘multi’) experimental series as training set.
**Figure S2** Differences in prediction ability attributed to presence of individual experimental series in the training set.
**Figure S3** Prediction accuracy (Prediction ability, divided by the square root of the heritability).

## Data Availability

Data of series 1–4 were presented earlier. Data and data access are documented for experimental series 1 in Gogna *et al*. (2022) and for experimental series 2–4 in Zhao *et al*. (2021). Data of experimental series 5–8 are not available publicly as they are trade secrets of the respective breeding companies. They are archived at the IPK.
