# Supplementary material for: Breaking down data silos across companies to train genome‐wide predictions: A feasibility study in wheat
Source: Plant Biotechnol J. 2025 Apr 20;23(7):2704–19. doi: 10.1111/pbi.70095 (PMC12205884; doi:10.1111/pbi.70095)
Supplement: Supplementary file 1 — Table S1 Number of overlapping genotypes between experimental series. Figure S1 Comparing prediction abilities using only one (‘single’) versus multiple (‘multi’) experimental series as training set. Figure S2 Differences in prediction ability attributed to presence of individual experimental series in the training set. Figure S3 Prediction accuracy (Prediction ability, divided by the square root of the heritability). [file PBI-23-2704-s001.docx]

Supplementary Material of “Breaking down data silos across companies to train genome-wide predictions -- a feasibility study in wheat”

Moritz Lell^1^, Ulrike Avenhaus^2^, Jost Dörnte^3^, Wera Maria Eckhoff^4^, Tobias Eschholz^5^, Mario Gils^5^, Martin Kirchhoff^5^, Michael Koch^3^, Sonja Kollers^4^, Nina Pfeiffer^4^, Matthias Rapp^2^, Valentin Wimmer^4^, Markus Wolf^6^, Jochen Reif^1^, Yusheng Zhao^1^

1) Leibniz Institute for Plant Genetics and Crop Plant Research, Corrensstraße 3, 06466 Seeland, Germany

2) W. von Borries-Eckendorf GmbH & Co. KG, Hovedisser Str. 94, 33818 Leopoldshöhe, Germany

3) Deutsche Saatveredelung AG, Weissenburger Straße 5, 59557 Lippstadt, Germany

4) KWS SAAT SE & Co. KGaA, Grimsehlstr. 31, 37574 Einbeck, Germany

5) Nordsaat Saatzucht GmbH, Böhnshauser Str. 1, 38895 Langenstein, Germany

6) SU BIOTEC GmbH, Am Schwabeplan 6B, 60439 Gatersleben, Germany

# Supplementary Tables

**Supplementary Table 1**: Number of overlapping genotypes between experimental series. Empty cells in the upper diagonal of the tables come from heading date and plant height data being not available for series 3 and 4.

| **Trait** | **Experimental series** | | | | | | | |
| --- | --- | --- | --- | --- | --- | --- | --- | --- |
|  |  | **2** | **3** | **4** | **5** | **6** | **7** | **8** |
| **Heading date** | **1** | 2 |  |  | 0 | 3 | 1 | 1 |
|  | **2** |  |  |  | 1 | 3 | 3 | 2 |
|  | **5** |  |  |  |  | 5 | 5 | 4 |
|  | **6** |  |  |  |  |  | 9 | 5 |
|  | **7** |  |  |  |  |  |  | 5 |
| **Plant Height** | **1** | 2 |  |  | 0 | 3 | 1 | 1 |
|  | **2** |  |  |  | 1 | 3 | 3 | 2 |
|  | **5** |  |  |  |  | 5 | 5 | 4 |
|  | **6** |  |  |  |  |  | 9 | 5 |
|  | **7** |  |  |  |  |  |  | 5 |
| **Grain Yield** | **1** | 26 | 6 | 15 | 1 | 3 | 1 | 1 |
|  | **2** |  | 13 | 44 | 4 | 8 | 7 | 4 |
|  | **3** |  |  | 7 | 1 | 1 | 2 | 1 |
|  | **4** |  |  |  | 3 | 7 | 5 | 3 |
|  | **5** |  |  |  |  | 7 | 7 | 5 |
|  | **6** |  |  |  |  |  | 9 | 5 |
|  | **7** |  |  |  |  |  |  | 5 |

# Supplementary Figures


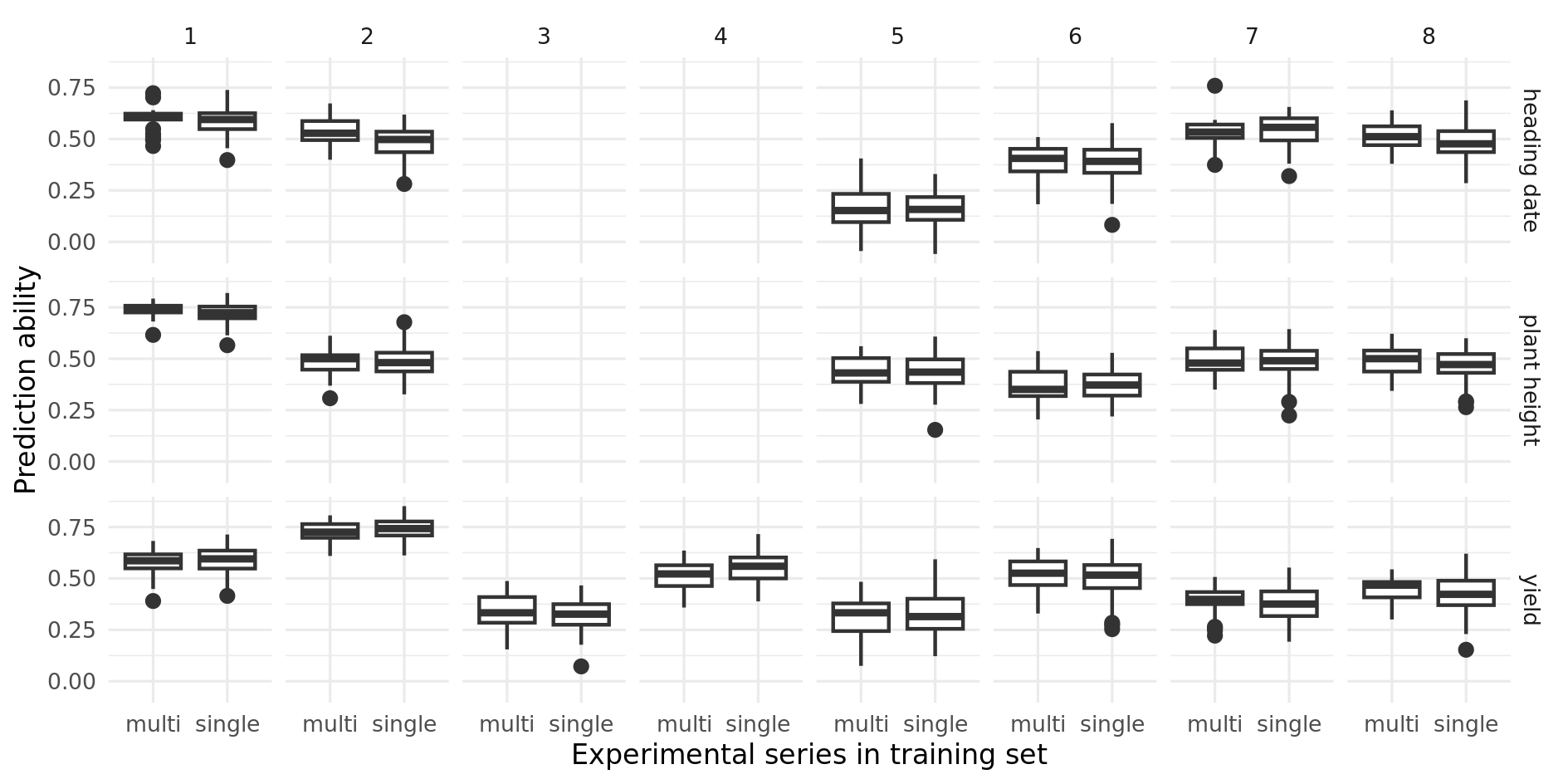


**Supplementary Figure 1:** Comparing prediction abilities using only one (“single”) versus multiple (“multi”) experimental series as training set. The training set sizes were constant at 800 genotypes. Each boxplot summarizes 25 replications. Each column of plots shows the results of using one experimental series as test set.


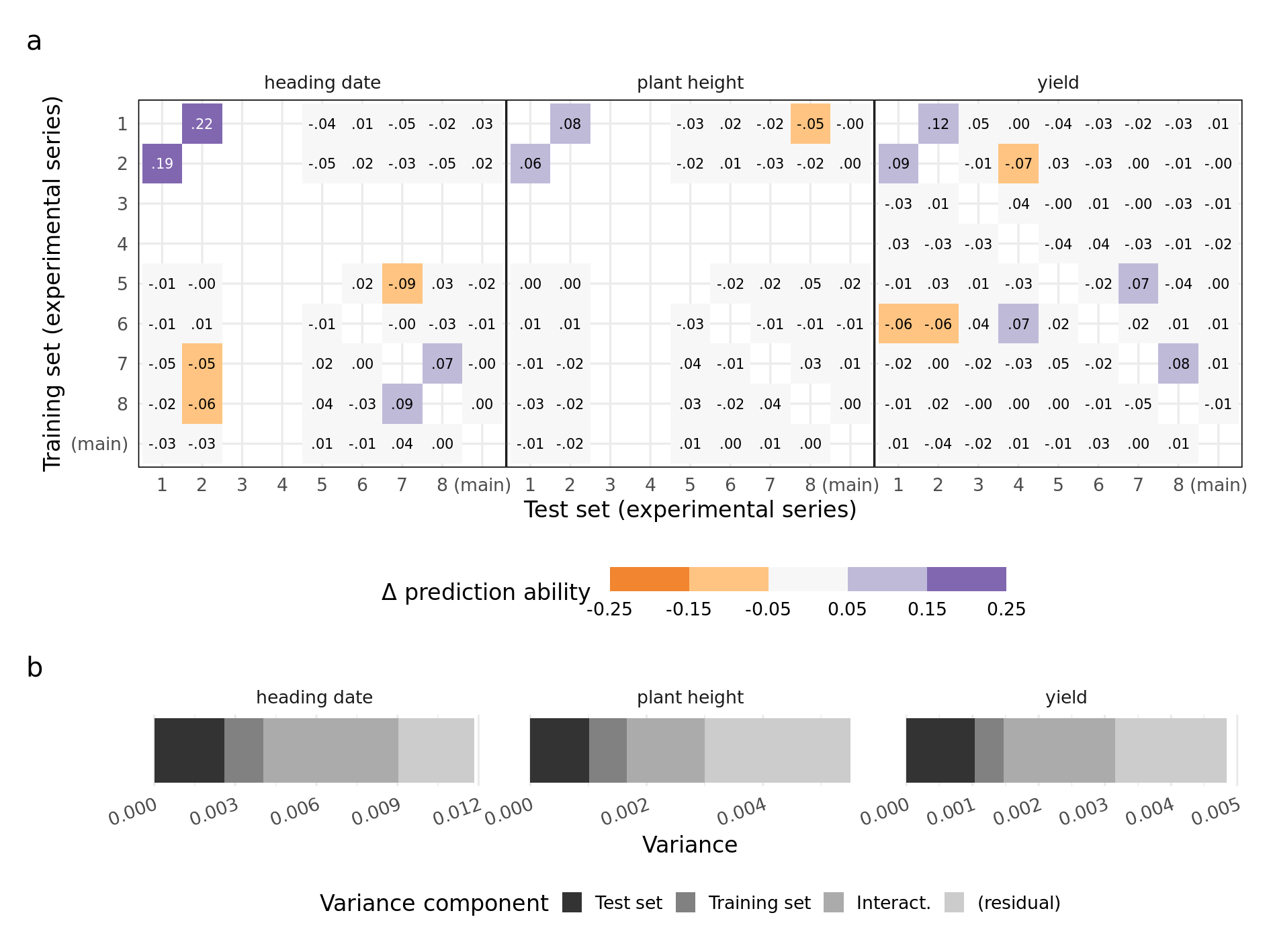


**Supplementary Figure 2:** Differences in prediction ability attributed to presence of individual experimental series in the training set. Prediction difference is respective to an average prediction ability given the test set and the training set size (empirical model, see Figure 6a). Differential prediction abilities are denoted by color and numbers.


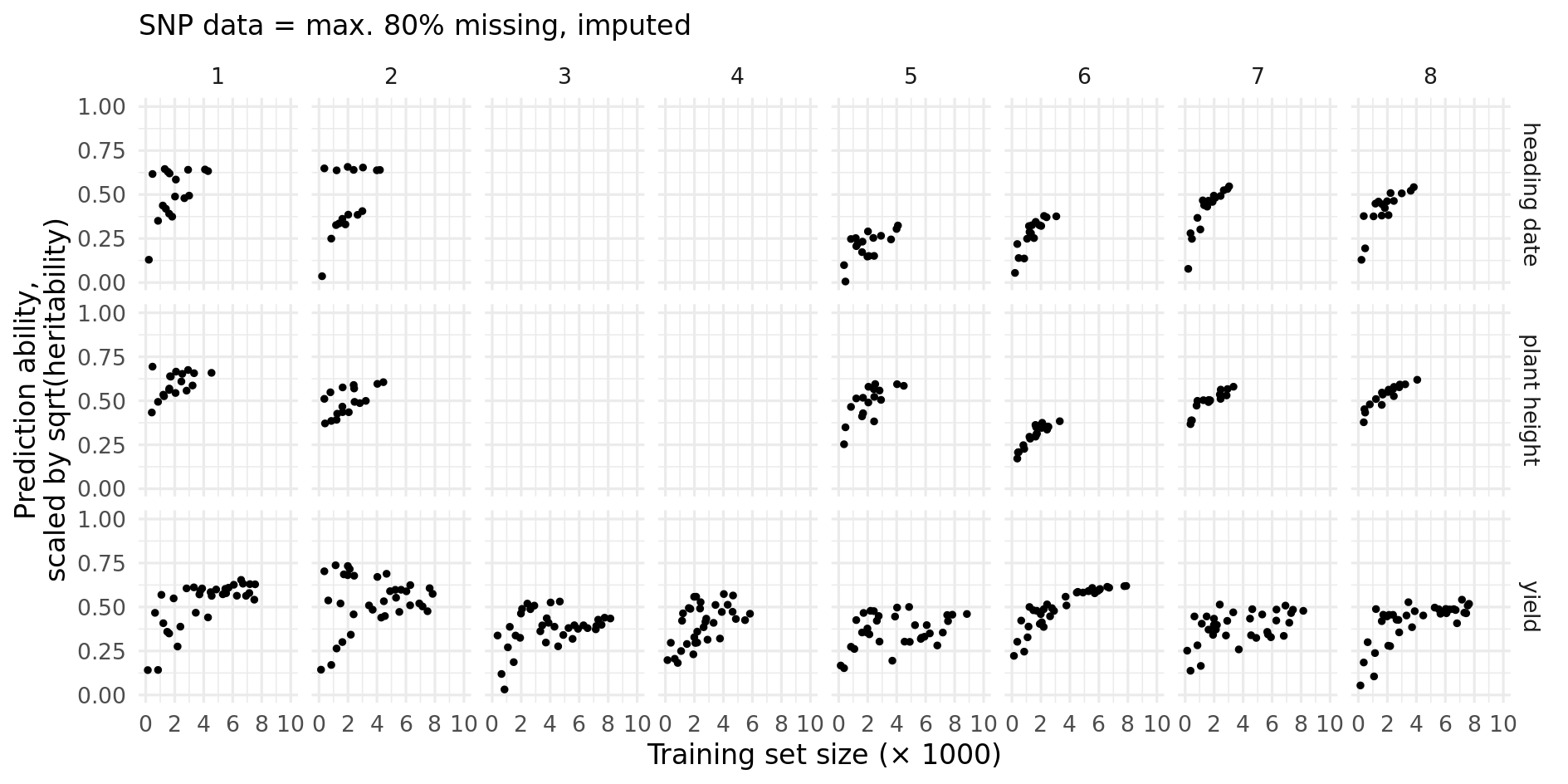


**Supplementary Figure 3:** Prediction accuracy (Prediction ability, divided by the square root of the heritability)
